# Sex‐ and age‐specific associations between cardiometabolic risk and white matter brain age in the UK Biobank cohort

**DOI:** 10.1002/hbm.25882

**Published:** 2022-04-23

**Authors:** Sivaniya Subramaniapillai, Sana Suri, Claudia Barth, Ivan I. Maximov, Irene Voldsbekk, Dennis van der Meer, Tiril P. Gurholt, Dani Beck, Bogdan Draganski, Ole A. Andreassen, Klaus P. Ebmeier, Lars T. Westlye, Ann‐Marie G. de Lange

**Affiliations:** ^1^ LREN, Centre for Research in Neurosciences, Department of Clinical Neurosciences Lausanne University Hospital (CHUV) and University of Lausanne Lausanne Switzerland; ^2^ Department of Psychology, Faculty of Science McGill University Montreal Quebec Canada; ^3^ Department of Psychology University of Oslo Oslo Norway; ^4^ Department of Psychiatry University of Oxford Oxford UK; ^5^ Wellcome Centre for Integrative Neuroimaging University of Oxford Oxford UK; ^6^ Norwegian Centre for Mental Disorders Research (NORMENT), Division of Mental Health and Addiction Oslo University Hospital and University of Oslo Oslo Norway; ^7^ Department of Psychiatric Research Diakonhjemmet Hospital Oslo Norway; ^8^ Department of Health and Functioning Western Norway University of Applied Sciences Bergen Norway; ^9^ School of Mental Health and Neuroscience, Faculty of Health Medicine and Life Sciences Maastricht University Maastricht The Netherlands; ^10^ Department of Neurology Max Planck Institute for Human Cognitive and Brain Sciences Leipzig Germany; ^11^ KG Jebsen Centre for Neurodevelopmental Disorders University of Oslo Oslo Norway

**Keywords:** *APOE* genetic risk, brain age, cardiometabolic health, menopause, sex differences

## Abstract

Cardiometabolic risk (CMR) factors are associated with accelerated brain aging and increased risk for sex‐dimorphic illnesses such as Alzheimer's disease (AD). Yet, it is unknown how CMRs interact with sex and apolipoprotein E‐*ϵ*4 (*APOE4*), a known genetic risk factor for AD, to influence brain age across different life stages. Using age prediction based on multi‐shell diffusion‐weighted imaging data in 21,308 UK Biobank participants, we investigated whether associations between white matter Brain Age Gap (BAG) and body mass index (BMI), waist‐to‐hip ratio (WHR), body fat percentage (BF%), and *APOE4* status varied (i) between males and females, (ii) according to age at menopause in females, and (iii) across different age groups in males and females. We report sex differences in associations between BAG and all three CMRs, with stronger positive associations among males compared to females. Independent of *APOE4* status, higher BAG (older brain age relative to chronological age) was associated with greater BMI, WHR, and BF% in males, whereas in females, higher BAG was associated with greater WHR, but not BMI and BF%. These divergent associations were most prominent within the oldest group of females (66–81 years), where greater BF% was linked to lower BAG. Earlier menopause transition was associated with higher BAG, but no interactions were found with CMRs. In conclusion, the findings point to sex‐ and age‐specific associations between CMRs and brain age. Incorporating sex as a factor of interest in studies addressing CMR may promote sex‐specific precision medicine, consequently improving health care for both males and females.

## Graphical abstract

In this study, we report sex differences in associations between white matter Brain Age Gap (BAG) and body mass index (BMI), waist‐to‐hip ratio (WHR), and body fat percentage (BF%). The findings point to sex‐ and age‐specific associations between cardiometabolic factors and brain age, highlighting the importance of considering sex as a variable of interest to promote precision medicine research.

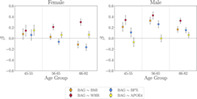

## 1 INTRODUCTION

Cardiometabolic risk (CMR) factors such as obesity are associated with adverse health outcomes including accelerated brain aging (Beck, de Lange, Pedersen, et al., 2021) and increased risk for Alzheimer's disease (AD; Livingston et al., 2020; Qiu & Fratiglioni, 2015). The impact of both CMRs and genetic risk for AD, such as apolipoprotein E‐*ϵ*4 (*APOE4*), are known to differ between males and females (Alqarni et al., 2021; Bretsky et al., 1999; Geerlings et al., 2010; Gerdts & Regitz‐Zagrosek, 2019; Neu et al., 2017; Schorr et al., 2018). Yet, it is unknown whether these risk factors interact in sex‐specific ways to influence brain health across different age periods or endocrine life stages. While males experience greater incidence and prevalence of cardiometabolic disease in early midlife, the greatest risk of cardiometabolic disease in females is observed up to 10 years later, coinciding with the menopausal transition (Dubnov et al., 2003; Maas & Appelman, 2010). Postmenopause, female *APOE4* carriers are also at greater AD risk than their male counterparts (Bretsky et al., 1999; Neu et al., 2017), but it is unknown whether a reduction in neuroprotective ovarian hormones, combined with an elevated cardiometabolic and genetic risk profile, may accelerate brain aging and subsequent AD risk in females compared to males. Investigating how CMRs interact with *APOE* genotype to influence the brain at different life stages, whether age‐ or menopause‐related, may thus clarify sex‐specific risk profiles for accelerated brain aging and illuminate critical periods for preventive interventions.

Poor cardiometabolic health is associated with changes in brain microvasculature, which may be reflected in magnetic resonance imaging (MRI)‐derived indices of white matter (WM) microstructure (Alfaro et al., 2018). CMRs such as adiposity, hypertension, smoking, and diabetes have been associated with lower fractional anisotropy (Birdsill et al., 2017; Mueller et al., 2011; Stanek et al., 2011), lower myelin and iron content (Trofimova et al., 2021), and greater WM hyperintensity (WMH) burden (Alqarni et al., 2021; Griffanti et al., 2018; Habes et al., 2018; Lampe et al., 2019; Raffield et al., 2016; Sachdev et al., 2009) in healthy older adults. Although females usually have higher volumes of WMH than males (de Leeuw et al., 2001; DeCarli et al., 2005; Fatemi et al., 2018; Sachdev et al., 2009; van den Heuvel et al., 2004), extensive literature indicates that males with CMRs (adiposity, hypertension, diabetes, and atherosclerosis) are more likely to develop WMH compared to females with similar levels of risk (Alqarni et al., 2021; Assareh et al., 2014; Filomena et al., 2015; Geerlings et al., 2010; Jongen et al., 2007). Hence, cardiometabolic health may influence WM microstructure differently in males and females, and risk profiles may also vary with age. Despite the consensus that high blood pressure and cholesterol levels are associated with accelerated brain aging and elevated dementia risk (Qiu et al., 2005; Solomon et al., 2009; van Vliet, 2012), the role of body fat is inconclusive (Fitzpatrick et al., 2009; Stewart et al., 2005). While mixed findings may be a result of selection or survivor bias (Heffernan et al., 2016; Jacobsen et al., 2021; Munafò et al., 2018; Salthouse, 2014), or variation in body fat indices used across studies (e.g., body mass index [BMI] versus waist‐to‐hip ratio [WHR] or body fat percentage [BF%]; Huxley et al., 2010; Lavie et al., 2012; Tchernof & Després, 2013; Tomiyama et al., 2016), they could also reflect a variable role of body composition throughout the lifespan. For example, one study found that BMI had a positive association with dementia risk when measured >20 years before dementia diagnosis, and a negative association when measured <10 years before dementia diagnosis (Kivimäki et al., 2018). Low BMI at later life stages may indicate frailty, sarcopenia (muscle loss), or preclinical dementia (Buchman et al., 2005; Hassan et al., 2019; Johnson et al., 2006; Subramaniapillai et al., 2021). Hence, while high BMI in mid‐adulthood may largely reflect obesity, higher BMI in senescence may reflect overall physical fitness or lack of degenerative diseases. Furthermore, body fat may act as a source of estrogen in postmenopausal females (Simpson, 2003), potentially protecting against WM decline (Klosinski et al., 2015). However, only a few studies have tried to disentangle the role of body fat composition in endocrine versus chronological aging (Sowers et al., 2007; Trikudanathan et al., 2013). Since risk profiles for adverse brain health may vary by sex across certain life stages, it is relevant to investigate specific age windows at which CMRs may have sex‐ and genotype‐specific effects on the brain.

One strategy for detecting atypical brain aging, particularly if it does not involve visible pathognomonic hallmarks of degenerative brain disease, is to use machine learning to predict an individual's age based on neuroimaging‐derived measures (Cole & Franke, 2017; Cole, Marioni, et al., 2019; Kaufmann et al., 2019). Brain Age Gap (BAG) provides a measure of deviation from expected age trajectories, and has been used to identify differences in patients with neurological and psychiatric disorders relative to healthy controls (Cole, Raffel, et al., 2019; Franke & Gaser, 2019; Han et al., 2020; Kaufmann et al., 2019; Rokicki et al., 2020; Tønnesen et al., 2020), as well as predicting future dementia risk (Wang et al., 2019) and prognosis (Biondo et al., 2020; Franke & Gaser, 2012; Gaser et al., 2013; Löwe et al., 2016). While individual variation in BAG reflects a combination of genetic and environmental factors (Elliott et al., 2019; Kaufmann et al., 2019; Vidal‐Piñeiro et al., 2021), clinical studies indicate that an older “brain age” relative to what is expected for an individual's chronological age (i.e., positive BAG) may in part reflect accelerated neural aging processes (Cole, Raffel, et al., 2019; Han et al., 2020; Kaufmann et al., 2019; Kolenic et al., 2018; Rokicki et al., 2020; Tønnesen et al., 2020; van Gestel et al., 2019). Positive BAG values have also been associated with negative outcomes in population‐based studies, including cardiovascular risk, cognitive impairments, and dementia risk (Biondo et al., 2021; de Lange, Anatürk, et al., 2020; Egorova et al., 2019; Franke & Gaser, 2012; Gaser et al., 2013; Kolbeinsson et al., 2020; Löwe et al., 2016; Wang et al., 2019). Previous studies have shown accurate age prediction based on diffusion‐weighted imaging measures (Beck, de Lange, Maximov, et al., 2021; Cole, 2020; Richard et al., 2018; Voldsbekk et al., 2021), as well as associations between WM BAG and CMRs (Beck, de Lange, Alnaes, et al., 2021; Beck, de Lange, Pedersen, et al., 2021). However, these previous studies did not assess sex‐specific effects, or whether CMRs interact with *APOE* genotype to influence WM BAG during certain life phases.

In this study, we examined the associations between WM BAG and key CMRs, including BMI, WHR, and BF% (Bowman et al., 2017; Bradbury et al., 2017; Ul‐Haq et al., 2014), and *APOE4* status in males (*N* = 10,605) and females (*N* = 10,703). We further assessed whether these risk factors had salient effects in middle age (44–55 years) and different stages of older adulthood (56–65 years and 66–82 years) (Grady et al., 2006; Subramaniapillai, Rajagopal, et al., 2019; Subramaniapillai, Rajah, et al., 2019). Participants' brain ages were computed using a prediction model based on WM measures derived from three diffusion imaging modes: diffusion tensor imaging (DTI) (Basser et al., 1994), diffusional kurtosis imaging (DKI) (Jensen et al., 2005), and WM tract integrity (WMTI) (Fieremans et al., 2011). While DTI is commonly used to estimate WM indices that are highly sensitive to age (Beck, de Lange, Maximov, et al., 2021; Krogsrud et al., 2016; Storsve et al., 2016; Westlye et al., 2010), biophysical diffusion models such as WMTI (Fieremans et al., 2011), which is derived from DKI (Jensen et al., 2005), may more accurately capture WM tissue structure complexity (Jelescu & Budde, 2017; Jensen et al., 2005), thus providing greater biological specificity (Novikov et al., 2018). Given previous work indicating structural and functional sex differences in the human brain (Armstrong et al., 2019; Kaczkurkin et al., 2019; Ritchie et al., 2018; Scheinost et al., 2015), the diffusion metrics were used as input features to three separate prediction models: (1) mixed sex, (2) female only, and (3) male only, in order to improve the accuracy of the sex‐specific analyses (Biskup et al., 2019; Cirillo et al., 2020; Kaufmann et al., 2019). We used linear regression models to assess whether associations between BAG and BMI, WHR, BF%, and *APOE* genotype varied (i) between males and females, (ii) according to age at menopause in females, and (iii) across different age groups in males and females.

## 2 METHODS AND MATERIALS

### 2.1 Sample characteristics

The initial sample was drawn from the UK Biobank cohort (www.ukbiobank.ac.uk), and included 39,232 participants with diffusion‐weighted imaging and demographic data. We excluded 3379 participants with diagnosed brain disorders based on ICD10 (chapter V and VI, field F; *mental and behavioral disorders*, including F00–F03 for AD and dementia, and F06.7 “Mild cognitive disorder,” and field G; *diseases of the nervous system*, including inflammatory and neurodegenerative diseases (except G55‐59; “Nerve, nerve root and plexus disorders”). Diagnostic details are provided in the UK Biobank online resources (http://biobank.ndph.ox.ac.uk/showcase/field.cgi?id=41270), and in the ICD10 diagnostic manual (https://www.who.int/classifications/icd/icdonlineversions). In addition, 113 participants were excluded based on MRI outliers (see Section 2.2), leaving a total of 35,740 participants with diffusion‐weighted imaging data that were included in the brain age models. Only participants with complete data on demographic factors, *APOE* genotype, BMI, WHR, and BF% from the MRI assessment time point were included in the subsequent analyses, yielding a final sample of 21,308 (male = 10,605, female = 10,703). Sample demographics are provided in Table 1. Sex of participants refers to binary data on biological sex acquired from the NHS registry at recruitment, but in some cases updated by the participant (see https://biobank.ndph.ox.ac.uk/showcase/field.cgi?id=31).

**TABLE 1:** Sample demographics

Variable		Male	Female	*p*‐value	Test
*N*		10,605	10,703		
Age	Mean ± SD	64.58 ± 7.65	63.12 ± 7.33	<.001	KW
	Range (years)	44.57–81.30	45.48–81.89		
Ethnic background	% White	97.33	97.39	<.001	*X* ^2^
	% Black	0.47	0.62		
	% Mixed	0.35	0.46		
	% Asian	1.31	0.63		
	% Chinese	0.20	0.30		
	% Other	0.34	0.61		
Education	% University/college degree	50.00	47.81	<.001	*X* ^2^
	% A levels or equivalent	12.08	15.00		
	% O levels/GCSE or equivalent	17.06	20.41		
	% NVQ or equivalent	11.32	6.67		
	% Professional qualification	3.92	5.56		
	% None of the above	5.62	4.56		
Assessment location (N)	Newcastle	1916	1999	.04	*X* ^2^
	Cheadle	7280	7178		
	Reading	1409	1526		
*APOE*4 status	% carrier	25.48	26.65	.05	*X* ^2^
	% noncarrier	74.52	73.35		
BMI	Mean ± SD	26.72 ± 3.60	25.65 ± 4.18	<.001	KW
	Range	16.38–39.95	14.55–39.99		
WHR	Mean ± SD	0.93 ± 0.06	0.81 ± 0.07	<.001	KW
	Range	0.53–1.26	0.60–1.16		
BF%	Mean ± SD	25.12 ± 5.47	35.57 ± 6.44	<.001	KW
	Range	5.20–42.50	7.40–57.5		

*Note*: Mean ± standard deviation (SD) and ranges for age, body mass index (BMI), waist‐to‐hip ratio (WHR), and body fat percentage (BF%), and % in each group for ethnic background, education, assessment location, and *APOE4* status.

Abbreviations: GCSE, general certificate of secondary education; KW, Kruskal–Wallis; NVQ, national vocational qualification; *X* ^2^, chi‐squared test.

### 2.2 MRI data acquisition and processing

A detailed overview of the UK Biobank data acquisition and protocols is available in (Alfaro‐Almagro et al., 2018; Miller et al., 2016). Briefly, we processed diffusion‐weighted imaging data using an optimized diffusion pipeline as described in detail in Maximov et al., 2019. We included metrics derived from DTI (Basser et al., 1994), DKI (Jensen et al., 2005), and WMTI (Fieremans et al., 2011) as input features in the age prediction models, as described in Voldsbekk et al., 2021. The metrics for each model are listed in Supporting Information (SI) Section 1. The metrics were extracted based on subject‐specific skeletonized images (Smith et al., 2006), and Johns Hopkins University (JHU) atlases for WM tracts (with 0 thresholding; Mori et al., 2005) were used to provide global mean values and regional measures for 12 tracts used in previous aging and development studies (Krogsrud et al., 2016; Storsve et al., 2016; Voldsbekk et al., 2021; Westlye et al., 2010); anterior thalamic radiation, corticospinal tract, cingulate gyrus, cingulum hippocampus, forceps major, forceps minor, inferior fronto‐occipital fasciculus, inferior longitudinal fasciculus, superior longitudinal fasciculus, uncinate fasciculus, superior longitudinal fasciculus temporal, and corpus callosum. The included diffusion MRI data passed tract‐based spatial statistics (TBSS) post‐processing quality control using the YTTRIUM algorithm (Maximov et al., 2021), and were residualized with respect to scanning site using linear models. To remove further outliers, participants with SD ± 4 on the global mean FA measure were excluded, yielding a final sample of 35,740 participants with MRI data (male = 16,909, female = 18,831). To optimize prediction accuracy, the full MRI sample was included in the brain age models (Section 2.3), while the subsequent analyses (Sections 2.5 and 2.6) included only participants with complete data on CMRs and *APOE* genotype (*N* = 21,308; Table 1).

### 2.3 Brain age prediction

We ran three age prediction models: (1) mixed sex, (2) female only, and (3) male only, to obtain sex‐specific BAG values (Biskup et al., 2019; Kaufmann et al., 2019) as well as general BAG estimates based on the mixed sample. The prediction models were run using the *XGBoost* regression algorithm (*eXtreme Gradient Boosting*; https://github.com/dmlc/xgboost). *XGboost* includes advanced regularization to reduce over‐fitting, and has shown superior performance in machine learning competitions (Chen & Guestrin, 2016). Parameters were tuned in a nested cross‐validation using five inner folds for grid search, and 10 outer folds for model validation. Feature importance rankings for each model were extracted using gain scores, which are calculated based on each feature's contribution to each tree in the model and thus indicate the relative contribution of each feature to the prediction. BAG values were calculated by subtracting chronological age from predicted brain age. The age and BAG distributions for each of the age prediction models are shown in SI Figure 1. To ensure that associations with the variables of interest were not driven by age‐dependence in the BAG estimations (Li et al., 2018; Liang et al., 2019), chronological age was regressed out of the BAG values before they were used in subsequent analyses (de Lange & Cole, 2020; Le et al., 2018). As a cross‐check, we performed a supplementary analysis for the mixed‐sex model where we used 10% of the data as a held‐out validation sample (*N* = 3574) for model optimization (grid search), and derived the best‐fit parameters to run a separate age prediction model in the rest of the sample (*N* = 32,166) with 10‐fold cross‐validation.

### 2.4 APOE genotyping

Participants' *APOE* genotype was extracted using the UK Biobank version 3 imputed data, which has been rigorously assessed for quality control by the UK Biobank genetics team (Bycroft et al., 2018). The two *APOE* single‐nucleotide polymorphisms—rs7412 and rs429358 (Lyall et al., 2016) were used to estimate *APOE* genotype. *APOE ϵ*4 status was labeled *carrier* for *ϵ*3/*ϵ*4 and *ϵ*4/*ϵ*4 combinations, and *noncarrier* for *ϵ*2/*ϵ*2, *ϵ*2/*ϵ*3, and *ϵ*3/*ϵ*3 combinations (Lyall et al., 2019). We removed participants with the homozygous *ϵ*2/*ϵ*4 allele combination due to its ambiguity with *ϵ*1/*ϵ*3 (Lyall et al., 2016; Wisdom et al., 2011). For more information on the genotyping process, refer to (Bycroft et al., 2018).

### 2.5 CMR factors: BMI, WHR, and BF%

**FIGURE 1:**
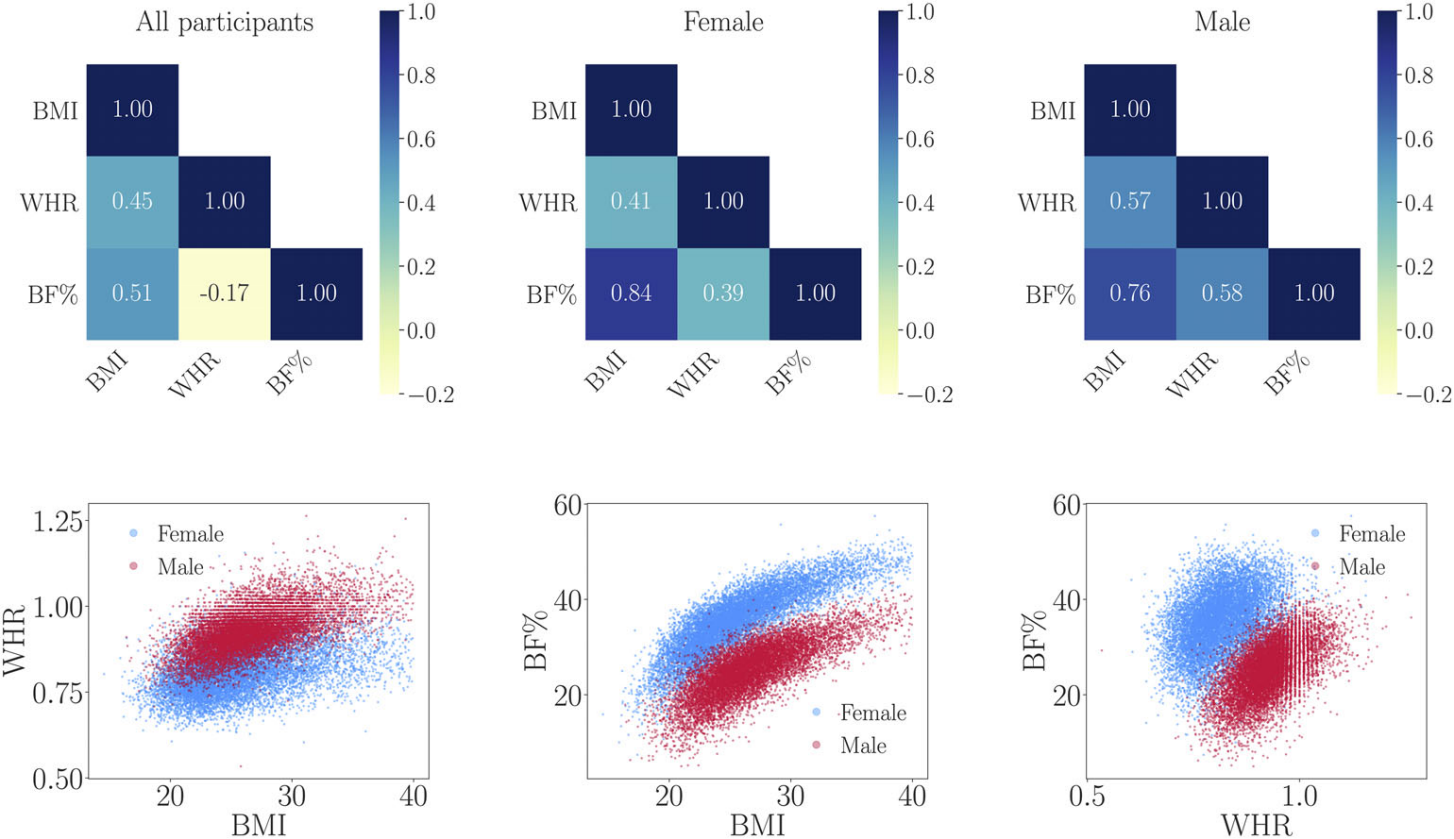
The matrices (top row) show the correlations (Pearson's coefficients) between BMI, WHR, and BF% for all participants, as well as females and males separately. The scatter plots (bottom row) show the correlations for all participants with males plotted in red and females in blue

CMRs included BMI (kg/m^2^), WHR (waist circumference/hip circumference), and BF% based on body composition by impedance measurement. All assessment procedures are described in detail in the UK Biobank protocol (Elliott & Peakman, 2008). As compared to BMI, which is a general measure of body adiposity, BF% distinguishes fat from muscles, while WHR is a more specific measure of abdominal obesity. Participants with BMI >40 were excluded (*N* = 196), since these values indicate morbid obesity and risk for serious health complications and comorbidities (Jarolimova et al., 2013; Schelbert, 2009). The correlations between BMI, WHR, and BF% are shown in Figure 1, indicating shared variance corresponding to previous studies (Chen et al., 2010; Myint et al., 2014; Ranasinghe et al., 2013; Ul‐Haq et al., 2014; Wiltink et al., 2013).

### 2.6 Categorizing groups based on (i) age at menopause and (ii) chronological age

To investigate whether associations of BAG with CMRs and *APOE* genotype varied according to age at menopause, we analyzed data from a subset of menopausal females who had complete information on age at menopause (*N* = 9693). Based on previous studies binning age at menopause in approximately 5‐year group bins (Gordon et al., 1978; Wasti et al., 1993), we applied similar age at menopause (i.e., Menopause Age Group) categories to our sample using the following bins: 40–45 years, 46–50 years, 51–55 years, and 56–62 years (Table 2). Since the average age of menopause is typically around 51.5 years and perimenopause lasts on average 4 years (Brinton et al., 2015; Harlow et al., 2012), we excluded females with age at menopause <40 years (*N* = 205) and >62 years (*N* = 4) from our female‐specific analyses to ensure that results were not driven by extreme values. We also excluded participants who had undergone a hysterectomy and/or oophorectomy (*N* = 714) before natural menopause, as these females often experience premature menopause associated with their surgery and may be at elevated risk of dementia (Rocca et al., 2007; total *N* of participants included = 8770). As a cross‐check, we repeated the interaction analyses including all participants. Distributions for BMI, WHR, and BF% within each Menopause Age Group are shown in SI Figure 2.

**TABLE 2:** Number of participants in each Menopause Age Group and each Age Group, separated by sex and with % of *APOE4* carriers in brackets

	Female	
*Menopause Age Group*	*N* (*APOE*4)	
40–45 years	986 (27.48%)	
46–50 years	2842 (26.39%)	
51–55 years	4059 (27.32%)	
56–62 years	883 (25.93%)	

	Female	Male
*Age Group*	*N* (*APOE*4)	*N* (*APOE*4)
45–55 years	1049 (29.26%)	1651 (26.95%)
56–65 years	4127 (27.16%)	3716 (25.61%)
66–82 years	3594 (25.90%)	5238 (24.91%)

To investigate whether effects of CMRs and *APOE* risk varied across different age groups, we categorized participants' ages (i.e., Age Group) using the following bins: 45–55 years, 56–65 years, and 66–82 years (Table 2). The bins were selected to take the full cohort age range into account, and to enable comparisons of effects in middle‐age and different stages of older adulthood in line with previous studies examining participants throughout the adult lifespan (Grady et al., 2006; Subramaniapillai, Rajagopal, et al., 2019; Subramaniapillai, Rajah, et al., 2019). Distributions for BMI, WHR, and BF% within each Age Group are shown in SI Figure 2.

To test if results were consistent between Menopause Age Group and Age Group bins and the continuous measures of these variables, we conducted supplementary analyses using the continuous variables of Age at Menopause and Age.

### 2.7 Statistical analyses

The statistical analyses were conducted using Python 3.7.6 and R version 3.5. All variables were standardized (subtracting the mean and dividing by the SD) before being entered into the analyses. *p*‐values were corrected for multiple comparisons using false discovery rate (FDR) correction (Benjamini & Hochberg, 1995). We report the *F*‐test significance of each main effect and each interaction, as *F* is useful for interpreting models containing categorical variables with more than two levels. The *F*‐statistics were generated in R by adding the *Anova* wrapper function to the linear model (*lm*) of interest.

We first determined whether there were sex differences in the effects of CMRs and *APOE* genotype on WM BAG, with the dependent variable representing BAG values based on the mixed‐sex age prediction model (*BAG*_ms_). In order to adjust for age‐dependence in CMRs and *APOE4* status, age was included as a covariate. The following *lm* was used for these analyses, with *x* representing each CMR (BMI, WHR, and BF%) or *APOE4* status, respectively:

(1) $${\mathrm{BAG}}_{\mathrm{ms}}∼x\times \mathrm{Sex}+\mathrm{Age}.$$

To test if interactions between sex and CMRs varied according to *APOE4* status, the following *lm* was used with *CMR* representing each risk factor (BMI, WHR, and BF%):

(2) $${\mathrm{BAG}}_{\mathrm{ms}}∼\mathrm{CMR}\times \mathrm{Sex}\times \text{APOE}+\mathrm{Age}.$$

We then used the BAG values from the female‐specific model to determine whether CMR and *APOE* effects on BAG varied according to age at menopause in females (Menopause Age Group). The following *lm* was used for these analyses, with *BAG*_ss_ representing BAG estimates based on the sex‐specific model, and *x* representing each CMR or *APOE4* status, respectively:

(3) $${\mathrm{BAG}}_{\mathrm{ss}}∼x\times \text{Menopause}\;\mathrm{Age}\;\text{Group}+\mathrm{Age}.$$

To test if interactions between Menopause Age Group and CMR varied according to *APOE* genotype, the following *lm* was used with *CMR* representing each risk factor:

(4) $${\mathrm{BAG}}_{\mathrm{ss}}∼\mathrm{CMR}\times \text{Menopause}\;\mathrm{Age}\;\text{Group}\times \text{APOE}+\mathrm{Age}.$$

To determine whether effects in females and males varied across age bins, we ran analyses within each sex assessing the interactions of CMRs, *APOE* genotype, and Age Group on BAG. Sex‐specific BAG values were used as dependent variables in the following *lm*, with *x* representing each CMR or *APOE4* status, respectively:

(5) $${\mathrm{BAG}}_{\mathrm{ss}}∼x\times \mathrm{Age}\;\text{Group}.$$

To test if interactions between Age Group and CMRs varied according to *APOE* genotype, the following *lm* was used with *CMR* representing each risk factor:

(6) $${\mathrm{BAG}}_{\mathrm{ss}}∼\mathrm{CMR}\times \mathrm{Age}\;\text{Group}\times \text{APOE}.$$

As a follow‐up, we ran the following *lm* within each of the Age Groups (for each sex) adjusting for effects of age within each Age Group bin:

(7) $${\mathrm{BAG}}_{\mathrm{ss}}∼\mathrm{CMR}\times \text{APOE}+\mathrm{Age}.$$

## 3 RESULTS

### 3.1 Brain age prediction

The accuracy of the age prediction models are shown in Table 3. SI Tables 1 and 2 depict the top 10 WM features for the mixed‐sex and sex‐specific age predictions, with the majority of the features overlapping between the three models. The model accuracy and prediction values were highly consistent when using a held‐out validation sample for model optimization, as shown in SI Table 3.

**TABLE 3:** Age prediction accuracy for the mixed‐sex and sex‐specific models, including average *R* ^2^, root mean square error (RMSE), mean absolute error (MAE), and correlations (*r*) between predicted and chronological age

Model	*R* ^2^	RMSE	MAE	*r* [95% CI]	*p*
Mixed (*N* = 35,740)	0.51 ± 0.009	5.27 ± 0.007	4.23 ± 0.005	0.72[0.71, 0.72]	<.0001
Male (*N* = 16,909)	0.50 ± 0.017	5.39 ± 0.115	4.34 ± 0.080	0.71[0.70, 0.72]	<.0001
Female (*N* = 18,831)	0.49 ± 0.015	5.28 ± 0.081	4.27 ± 0.070	0.69[0.69, 0.70]	<.0001

Abbreviation: CI, confidence interval.

### 3.2 Sex differences in effects of CMRs and APOE genotype on WM BAG

**FIGURE 2:**
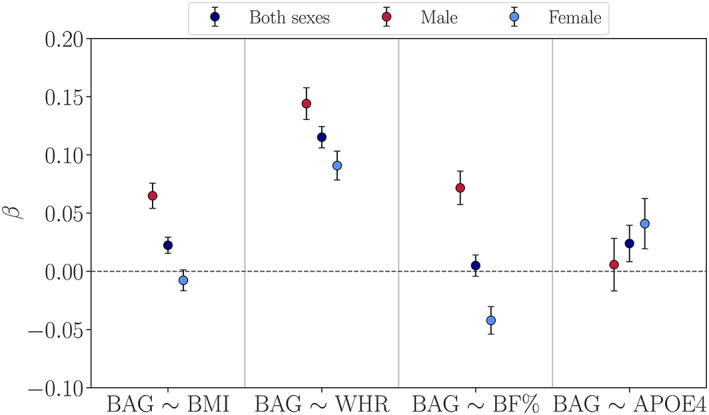
Associations between WM Brain Age Gap (BAG) and each cardiometabolic risk factor as well as *APOE4* status for both sexes, and for males and females separately. *β* (y‐axis) represents the beta value (slope) for each association, for example, a positive *β* value indicates an association between greater cardiometabolic measures or *APOE4* and higher BAG (older brain age relative to chronological age). The error bars represent standard errors on the *β*. BF%, body fat percentage; BMI, body mass index; WHR, waist‐to‐hip ratio

To assess sex differences in the associations between BAG and BMI, WHR, BF%, and *APOE4* status, we tested for sex‐interactions as described in Section 2.7. The analyses revealed significant interaction effects of *Sex* × *BMI*, *Sex* × *WHR*, and *Sex* × *BF%* on BAG, as shown in Table 4 and Figure 2. Greater WHR was associated with higher BAG (older brain age relative to chronological age) across sexes, but the effect was more prominent in males compared to females. BF% showed diverging associations, with higher values related to higher BAG in males and higher values related to lower BAG (younger brain age relative to chronological age) in females. Higher BMI values were associated with higher BAG in males, while no significant effect of BMI was seen in females. We observed no significant main effects of *APOE* genotype, and no significant interactions between *APOE* genotype and Sex or CMRs (see SI Table 4 for full results). Figure 2 shows the beta values for the BAG associations with each CMR and *APOE4* status for both sexes, and for males and females separately. For comparison, we produced the same plot using estimates based on the sex‐specific models (BAG values estimated relative to sex‐specific age trajectories), as shown in SI Figure 3. The results showed similar patterns, with associations between higher BAG and greater BMI, WHR, and BF% in males, and between higher BAG and greater WHR, but not BMI and BF%, in females.

**TABLE 4:** Sex differences in the associations between Brain Age Gap and body mass index (BMI), waist‐to‐hip‐ratio (WHR), body fat percentage (BF%), and *APOE4* status based on Formula (1) in Section 2.7

Interaction	*F*	*p*	*p*corr
Sex × BMI	29.15	6.76 × 10^−8^	1.35 × 10^−7^
Sex × WHR	11.45	7.2 × 10^−4^	9.56 × 10^−4^
Sex × BF%	43.37	4.65 × 10^−11^	1.86 × 10^−10^
Sex × APOE	0.93	0.34	0.34

*Note*: Degrees of freedom = (1, 21,303).

### 3.3 Sex‐ and age‐specific effects of CMRs and APOE genotype on WM BAG

To assess whether effects of CMRs and *APOE4* status varied according to age at menopause or across specific age periods, we next performed a series of regressions testing for interactions with Menopause Age Group in females and Age Group in males and females separately, as described in Sections 2.6 and 2.7.

#### 3.3.1 Effects of Menopause Age Group × CMRs and APOE genotype on WM BAG

In females, no interactions of CMR measures and *APOE4* status with Menopause Age Group were found, as shown in Table 5 (see SI Table 5 for full results). The results were consistent when using the continuous Age at Menopause variable, as shown in SI Table 6, and when including participants with age at menopause <40 and >62 years, hysterectomy and/or oophorectomy, as shown in SI Tables 7–9. Across models, there was a main effect of Menopause Age Group such that a lower age at menopause was associated with higher WM BAG (see SI Table 5 and SI Figure 2). This effect was consistent when using the continuous Age at Menopause variable in a regression model including hormone replacement therapy use, education, income, Townsend Deprivation Index, alcohol intake, physical activity, and number of childbirths in addition to age, BMI, and *APOE4* status as covariates (*β* = −0.036 ± 0.010, *p* = 1.40 × 10^−4^; see SI Section 6 for details about the covariates).

**TABLE 5:** The interaction between Menopause Age Group and body mass index (BMI), waist‐to‐hip‐ratio (WHR), body fat percentage (BF%), and *APOE4* status on Brain Age Gap in females (Formula 3, Section 2.7)

Interaction	*F*	*p*	*p*corr
BMI × Menopause Age Group	2.00	.11	.22
WHR × Menopause Age Group	1.50	.21	.28
BF% × Menopause Age Group	2.08	.10	.22
APOE × Menopause Age Group	0.73	.53	.53

*Note*: Degrees of freedom = (3, 8761).

#### 3.3.2 Effects of age group × CMRs and APOE genotype on WM BAG

**FIGURE 3:**
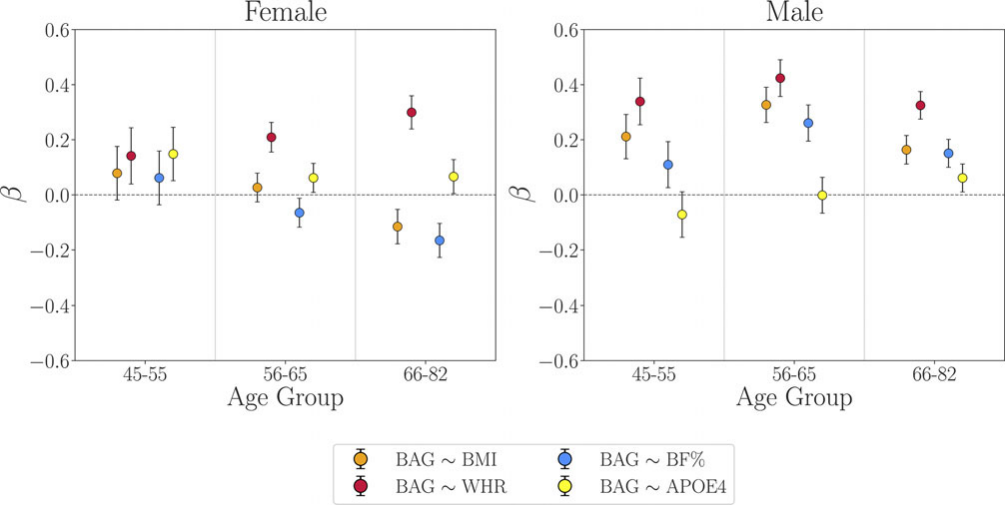
Associations between WM BAG and cardiometabolic risk factors as well as *APOE4* status within each Age Group bin (Formula 7, Section 2.7). *β* (y‐axis) represents the beta value (slope) for each association. The error bars represent standard errors on the *β*. BF%, body fat percentage; BMI, body mass index; WHR, waist‐to‐hip ratio

To assess whether effects of CMRs and *APOE4* status varied across specific age periods, we applied regressions including interaction terms with Age Group as described in Formula (5), Section 2.7. For both females and males, no significant effects were found for Age Group × CMR measures/*APOE4* status on BAG (Table 6 and SI Table 10), indicating that the BAG associations with each CMR did not vary significantly between age groups. The results were consistent when using the continuous Age variable (SI Table 11). Figure 3 shows the associations between BAG and each CMR within each Age Group, and SI Figure 5 shows the associations grouped by *APOE* genotype.

**TABLE 6:** The interaction between Age Group and BMI, WHR, BF%, and *APOE* genotype on Brain Age Gap (Formula 5, Section 2.7)

	Female			Male		
Interaction	*F*	*p*	*p*corr	*F*	*p*	*p*corr
BMI × Age Group	1.96	.14	.25	2.33	.10	.39
WHR × Age Group	1.68	.19	.25	0.72	.49	.49
BF% × Age Group	1.76	.17	.25	1.27	.28	.49
APOE × Age Group	0.31	.73	.73	0.87	.42	.49

*Note*: Degrees of freedom = (2, 8764) and (2, 10,599) for females and males, respectively.

While the BAG associations with each CMR were not significantly different between age groups (Table 6), the follow‐up regression analyses within each age group indicated that the divergence between the associations with WHR versus BMI/BF% increased with older age in females (Figure 3; see SI Tables 12 and 13 for full results). As a post hoc test, we estimated the differences between the WHR versus BMI/BF% associations for females within each age group using a *Z* test for correlated samples, as described in SI Section 7. The results confirmed that the divergence between the BAG associations with WHR versus BMI/BF% increased with age, and was most prominent in the oldest age group (SI Table 14).

## 4 DISCUSSION

This study investigated whether associations between BAG and CMRs and *APOE* genotype varied (i) between males and females, (ii) according to age at menopause in females, and (iii) across different age groups in males and females. In summary, the results showed sex differences in associations between BAG and all three CMRs, with stronger positive associations among males compared to females. Higher BAG (older brain age relative to chronological age) was associated with greater BMI, WHR, and BF% in males, whereas in females, higher BAG was associated with greater WHR, but not BMI and BF%. Earlier age at menopause was linked to higher BAG in females, but no interactions were found between age at menopause and CMRs. While none of the associations between BAG and each CMR were significantly different between age groups, follow‐up analyses indicated that the divergence between the WHR and BMI/BF% associations observed in females was most prominent within the oldest age group (66–81 years). *APOE4* status showed no significant main effects on BAG, no age‐ or age at menopause‐specific effects, and no significant interactions with CMRs. The findings demonstrate sex‐specific associations between body fat composition and brain age, emphasizing the importance of analyzing males and females separately in studies addressing CMR in aging.

The analyses including sex as an interaction term showed that all three CMRs were associated with higher BAG in males relative to females. These findings support a recent study, which revealed that males may be more vulnerable than females to WM brain aging in the presence of greater BMI (Alqarni et al., 2021) and, more broadly, greater cardiometabolic burden (Assareh et al., 2014; Filomena et al., 2015; Geerlings et al., 2010; Jongen et al., 2007). Our findings may shed some light on sex differences in the timing of cardiometabolic disease, emerging on average earlier in males than females (Maas & Appelman, 2010). Unlike females, it is possible that the combined effect of all three CMRs on males' higher BAG as early as midlife may, in turn, be associated with their earlier cardiometabolic disease risk. However, these measures of body adiposity may also be more sensitive to males (Foret et al., 2021), while other unexplored CMRs, such as hypertension, may be more robust in capturing females' health risk earlier in adulthood (Gilsanz et al., 2017; Wei et al., 2017). Furthermore, sex differences in adipose fat distribution may differentially contribute to brain age, with males more likely having fat distributed in the visceral adipose tissue surrounding the abdominal organs, while females tend to have more subcutaneous adipose tissue (Bredella, 2017; Chang et al., 2018). Compared to the latter, greater visceral adipose fat distribution is associated with elevated risk of cardiometabolic disease. In females, differential effects of BF% and BMI compared to WHR were observed, with divergent associations particularly prominent in the oldest age group (66–82 years) where lower BF% was linked to higher BAG. This could reflect that lower BF% in older females may be an indicator of frailty and preclinical dementia, and/or indicate a protective role of certain sources of adipose fat in females at later ages (Buchman et al., 2005; Johnson et al., 2006; Klosinski et al., 2015; Subramaniapillai et al., 2021). While our results showed that earlier menopause transition was associated with greater BAG, we found no significant interactions between age at menopause and CMRs. Further longitudinal work is required to clarify the role of adipose tissue in female brain health, and how it may relate to sex‐specific factors including the menopausal transition.

The evidence for the role of endogenous estrogen exposure in neurodegeneration and AD is controversial, with some studies reporting an association between shorter reproductive span and greater dementia risk (e.g., Gilsanz et al., 2019), while others have found that a longer reproductive span (i.e., later age of menopause) did not confer protective effects (e.g., Geerlings et al., 2001; Mosconi et al., 2018; Najar et al., 2020). Some studies have reported genotype‐specific effects, with a longer reproductive span conferring greater risk in *APOE4* carriers (Geerlings et al., 2001), and older brain age linked to greater estradiol levels in *APOE4* carriers and lower levels in noncarriers (de Lange, Barth, et al., 2020). These studies point to a possible modulatory role of estrogen exposure (Barth & de Lange, 2020), typically having beneficial effects, but potentially becoming neurotoxic in the context of greater AD pathology (Jack Jr et al., 2010) or diseased cell populations (i.e., “healthy cell” hypothesis; Brinton, 2005). While future studies including detailed data about the menopausal transition (i.e., pre‐, peri, postmenopause) as well as specific measures of AD‐related brain pathology (Rahman et al., 2020) are required to test these hypotheses, our findings showed a small but significant association between earlier menopause transition and higher WM BAG in the current cohort, in line with studies linking a shorter reproductive span to risk for neurodegeneration (Fox et al., 2013; Geerlings et al., 2001; Gilsanz et al., 2019; Mishra & Brinton, 2018; Schelbaum et al., 2021; Scheyer et al., 2018).

Unlike females, in which menses cessation is a marker of menopause status, males have a more gradual endocrine aging process, with no significant marker for age at andropause. Although low levels of body fat in older age may be an indicator of frailty in both sexes (Buchman et al., 2005; Johnson et al., 2006), our results show that in this sample, greater WHR, BMI, and BF% were consistently linked to higher BAG in males. This corresponds with previous studies reporting negative effects of higher body fat levels on brain health in males across midlife and older ages (Dekkers et al., 2019; Taki et al., 2008). The male endocrine aging process typically involves gradual declines in testosterone levels. Greater adipose tissue may increase levels of aromatase, an enzyme that converts testosterone to estrogen, which in males may be associated with their accelerated endocrine and consequently, brain aging process (Blouin et al., 2006; Meyer et al., 2011; Vosberg et al., 2021). Therefore, while greater adipose tissue can potentially act as a source of estrogen in postmenopausal females, it can be detrimental to males, in whom this may result in reduced testosterone levels (see Vosberg et al., 2021 demonstrating that the genetic architecture of testosterone contributes to sex differences in cardiometabolic traits in the UK Biobank). Importantly, increasing evidence points to the role of sex hormones in mediating cerebrovascular function, in which dysregulation is linked to cerebrovascular diseases, cognitive impairment, and dementia (see reviews by Gannon et al., 2019; Robison et al., 2019). Further research on the links between sex hormones and cardiometabolic factors in endocrine aging may help inform sex‐specific health interventions for both males and females.

No significant effects were found for *APOE* genotype. This is in line with our previous study showing no effects of *APOE4* status or polygenic risk for AD on gray‐matter based brain age in the UK Biobank sample (de Lange, Barth, et al., 2020), as well as a recent UK Biobank study showing that *APOE4* genotype was associated with WM hyperintensities, but not with FA or MD in WM tracts (Lyall et al., 2020). While our age prediction was based on several diffusion models known to be sensitive to WM aging (Beck, de Lange, Maximov, et al., 2021; Jelescu & Budde, 2017; Jensen et al., 2005), it is possible that specific estimates of WM hyperintensities could yield *APOE*‐sensitive CMR associations. Furthermore, a recent UK Biobank study revealed region‐ and metric‐specific effects of age and sex on WM microstructure (Lawrence et al., 2021). Although age prediction models combine a rich variety of WM characteristics into single estimates, global BAG estimates do not provide specific information about regional WM connections. Hence, future studies may aim to investigate regional and diffusion metric‐specific estimates of brain aging in relation to *APOE* genotype and CMRs. Modality‐specific BAG estimates are also relevant for identifying differences in brain tissue affected by a specific condition or disease (Beck, de Lange, Pedersen, et al., 2021; Cole, 2020; de Lange, Anatürk, et al., 2020; Rokicki et al., 2020). For example, one of our previous studies found that BMI interacted with AD risk to influence gray‐matter based BAG, such that females with greater AD risk benefited more from a higher BMI (Subramaniapillai et al., 2021). While the current study focused on WM measures given their susceptibility to CMRs, future studies may aim to include several brain modalities to directly compare sex‐ and age‐specific effects. More detailed measures of fat distribution obtained with body MRI (Beck, de Lange, Alnaes, et al., 2021; Gurholt et al., 2021; Leinhard et al., 2008; Linge et al., 2018) may also clarify the divergent associations observed in females, and provide a more complete understanding of adipose tissue distribution in relation to cardiometabolic disease (Linge et al., 2018; Linge et al., 2019), AD risk (Diehl‐Wiesenecker et al., 2015), and endocrine aging processes (El Khoudary et al., 2015). Due to sample size restrictions in relation to our study goals of determining sex‐ and age‐specific effects, we could not currently probe body MRI in our subgroups, but ongoing data collection of this measure from UK Biobank participants will render future analyses of these measures feasible.

Although the large UK Biobank cohort enabled us to investigate whether effects were sensitive to specific age‐ or age at menopause periods, the sample sizes were limited by probing variables with different subgroups (i.e., dividing our sample across sex, *APOE* genotype, and Menopause/Age Group levels). However, our sub‐group samples (*n* >250) are still large compared to the majority of previous studies investigating sex‐ or age‐specific effects on brain structure (Szucs & Ioannidis, 2020). The results also highlight potential causes of mixed findings in the literature: variations in associations reported across studies could be due to not separating analyses by sex (see Figure 2), investigating samples with different age ranges (see Figure 3 and SI Table 8), and/or the use of different CMRs. As participant re‐testing of the UK Biobank baseline cohort is actively underway, our future work will aim to integrate longitudinal investigations of brain age, as the current cross‐sectional analyses prevent any conclusions about causality. Longitudinal designs may also enable differentiation between age‐specific effects and effects that emerge as a result of a selective attrition or survival bias (Heffernan et al., 2016; Jacobsen et al., 2021; Munafò et al., 2018; Salthouse, 2014). While UK Biobank provides an excellent resource of open‐access population health data, the cohort is homogeneous with regard to ethnic background and education, and characterized by a “healthy volunteer effect” (Fry et al., 2017), indicating that it is not representative of the general population (Keyes & Westreich, 2019). Although the current results may not generalize to populations beyond those represented in this cohort, our findings may prompt further study into sex‐ and age‐specific effects of CMR as well as endocrine aging. Lastly, since neural aging processes are multi‐factorial, single risk factors can only explain parts of the individual variation. Hence, future studies may aim to go beyond investigating risk factors in isolation and adopt approaches that can model complex relationships between a variety of gene–environment interactions and brain health in aging (Mulugeta et al., 2021; Wang et al., 2020).

In conclusion, this study demonstrates notable sex differences in associations between body fat indices and WM brain age, underlining the importance of stratifying samples by sex in population‐based and clinical studies (Clayton, 2018; Ewelina et al., 2020; Ferretti et al., 2018; Miller et al., 2017; Shansky & Murphy, 2021; Shansky & Woolley, 2016). Independent of cardiometabolic profile, earlier menopause transition was associated with higher BAG in females. Hence, considering effects of both chronological and endocrine aging may increase our understanding of sex‐specific brain aging trajectories and disease prevalence (Jacobs & Goldstein, 2018; Taylor et al., 2019). Given the historical lack of research into sex‐specific influences on brain health and disease (Cirillo et al., 2020; de Lange, Jacobs, & Galea, 2020; Ferretti & Santuccione Chadha, 2021; Taylor et al., 2019), future studies incorporating sex as a variable of interest may provide valuable contributions to precision medicine research, consequently improving health care for both males and females.

## Supporting information


Data S1 Supporting Information

## Data Availability

The data that support the findings of this study are available through the UK Biobank data access procedures (https://www.ukbiobank.ac.uk/researchers). Code for running the age prediction models is available at https://github.com/amdelange/brainage_women.
