# Supplementary material for: Sex‐ and age‐specific associations between cardiometabolic risk and white matter brain age in the UK Biobank cohort
Source: Hum Brain Mapp. 2022 Apr 23;43(12):3759–74. doi: 10.1002/hbm.25882 (PMC9294301; doi:10.1002/hbm.25882)
Supplement: Supplementary file 1 — Data S1 Supporting Information [file HBM-43-3759-s001.pdf]

Supplementary Information (SI)

1. Feature importance ranking for the age prediction models

The age and BAG distributions for each of the age prediction models are shown in SI Figure 1.

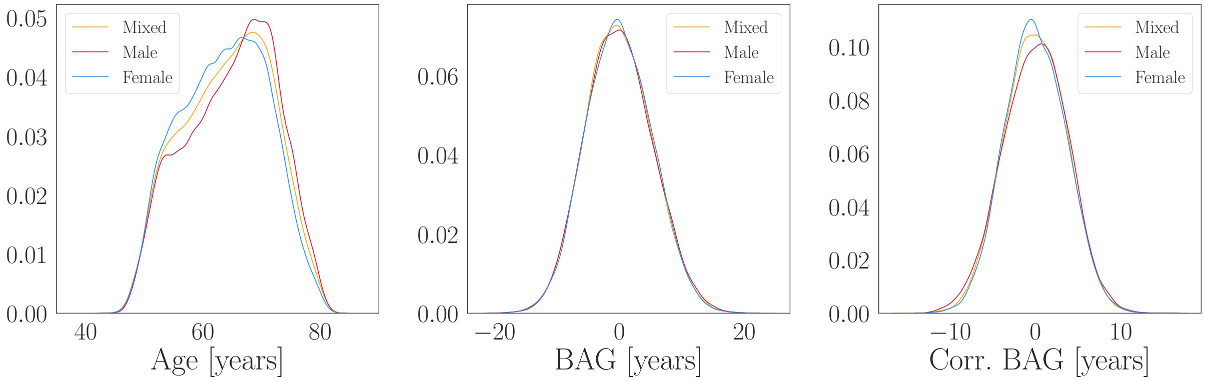

SI Figure 1: Age distributions (left plot), uncorrected brain age gap (BAG) distributions (middle plot), and age-corrected BAG distributions (right plot) for the sex-specific models. The distributions are normalised to have the same area, and the y-axes represent the density.

Metrics derived from DTI, DKI, and WMTI were used as input features in the age prediction models. The DTI metrics included mean diffusivity (MD), fractional anisotropy (FA), axial diffusivity (AD), and radial diffusivity (RD) [1]. The DKI metrics included mean kurtosis (MK), axial kurtosis (AK), and radial kurtosis (RK) [2]. WMTI metrics included axonal water fraction (AWF), extra-axonal axial diffusivity (axEAD), and extra-axonal radial diffusivity (radEAD) [3]. The feature importance rankings for each model are shown in SI Tables 1 and 2.

SI Table 1: Feature importance ranking for the sex-specific age prediction models, with *gain* for the top 12 features. The gain score indicates the relative contribution of the corresponding feature to the prediction model, calculated based on each feature's contribution for each tree in the model. A higher value implies that the feature was more important for generating the prediction.

Female-specific model		Male-specific model	
**Feature**	**Gain**	**Feature**	**Gain**
DTI FA - *FMIN*	13236.87962	DTI FA - *FMIN*	11254.45109
DTI FA - *IFOFL*	4291.27612	DTI MD - *ATR(r)*	4924.371643
WMTI radEAD - *IFOF(r)*	4177.059698	DTI FA - *IFOF(l)*	2340.885869
DTI RD - *ATR(l)*	3059.492715	DTI MD - *ATR(l)*	1839.073397
WMTI radEAD - *ATR(l)*	2706.212213	DTI AD - *CC*	1733.398119
DTI MD - *ATR(r)*	2287.285874	WMTI axEAD - *CC*	1589.52717
WMTI radEAD - *IFOF(l)*	1712.22425	WMTI radEAD - *IFOF(r)*	1319.972876
WMTI axEAD - *CC*	1643.873903	DKI AK - *ATR(r)*	1264.304262
DTI MD - *CST(l)*	1543.770175	WMTI radEAD - *ATR(l)*	1251.982447
DTI AD - *CC*	1329.163254	DKI RK - *CC*	1171.360486

Abbreviations: l = left, r = right. For **metrics**, see SI Section 1. **Diffusion models:** DTI = diffusion tensor imaging; DKI = diffusion kurtosis imaging; WMTI = white matter tract integrity. **Tracts:** ATR = anterior thalamic radiation; CST = corticospinal tract; CG = cingulate gyrus; CING = cingulum; FMAJ = forceps major; FMIN = forceps minor; IFOF = inferior fronto-occipital fasciculus; ILF = inferior longitudinal fasciculus; SLF = superior longitudinal fasciculus; UF = uncinate fasciculus; SLFT = superior longitudinal fasciculus temporal; CC = corpus callosum.

SI Table 2: Feature importance ranking for the age prediction model including both male and female participants, with *gain* for the top 10 features. The gain score indicates the relative contribution of the corresponding feature to the prediction model, calculated based on each feature's contribution for each tree in the model. A higher value implies that the feature was more important for generating the prediction.

**Feature**	**Gain**
DTI FA - *FMIN*	4412.278005
DTI FA - *IFOF(l)*	1568.794943
DTI MD - *ATR(r)*	1366.528915
DTI MD - *ATR(l)*	1418.28781
WMTI radEAD - *ATR(l)*	1310.913675
WMTI axEAD - *CC*	1051.857987
WMTI radEAD - *IFOF(r)*	862.9180985
DKI AK - *ATR(r)*	619.158944
DTI AD - *CC*	602.4423093
DTI AD - *ATR(r)*	548.4075138
DTI RD - *ATR(l)*	501.0781588

Abbreviations: l = left, r = right. For diffusion models, metrics, and tracts, see SI Table 1 notes.

SI Table 3: Model accuracy for the mixed-sex model including average R^2, root mean square error (RMSE), mean absolute error (MAE), and correlations (r) between predicted and chronological age, when using a held-out validation sample (N = 3,574) for model optimisation, and running the age prediction model with 10-fold cross validation in the rest of the sample (N = 32,166) to avoid data leakage. Best estimators based on the held-out validation set: learning rate = 0.05, max depth = 5, n estimators = 180. When comparing the estimates based on the main approach (manuscript Section 2.3.) and the current approach, the correlation between the values were r = 0.97 [0.97, 0.97] for predicted age, and r = 0.97 [0.97, 0.97] for brain age gap. CI = confidence interval.

Model	R^2	RMSE	MAE	r [95% CI]	p
Mixed (N = 32,166)	0.50 ± 0.013	5.33 ± 0.007	4.30 ± 0.007	$0.71[0.70, 0.71]$	<0.0001

2. CMR distributions within each Menopause Age Group and Age group

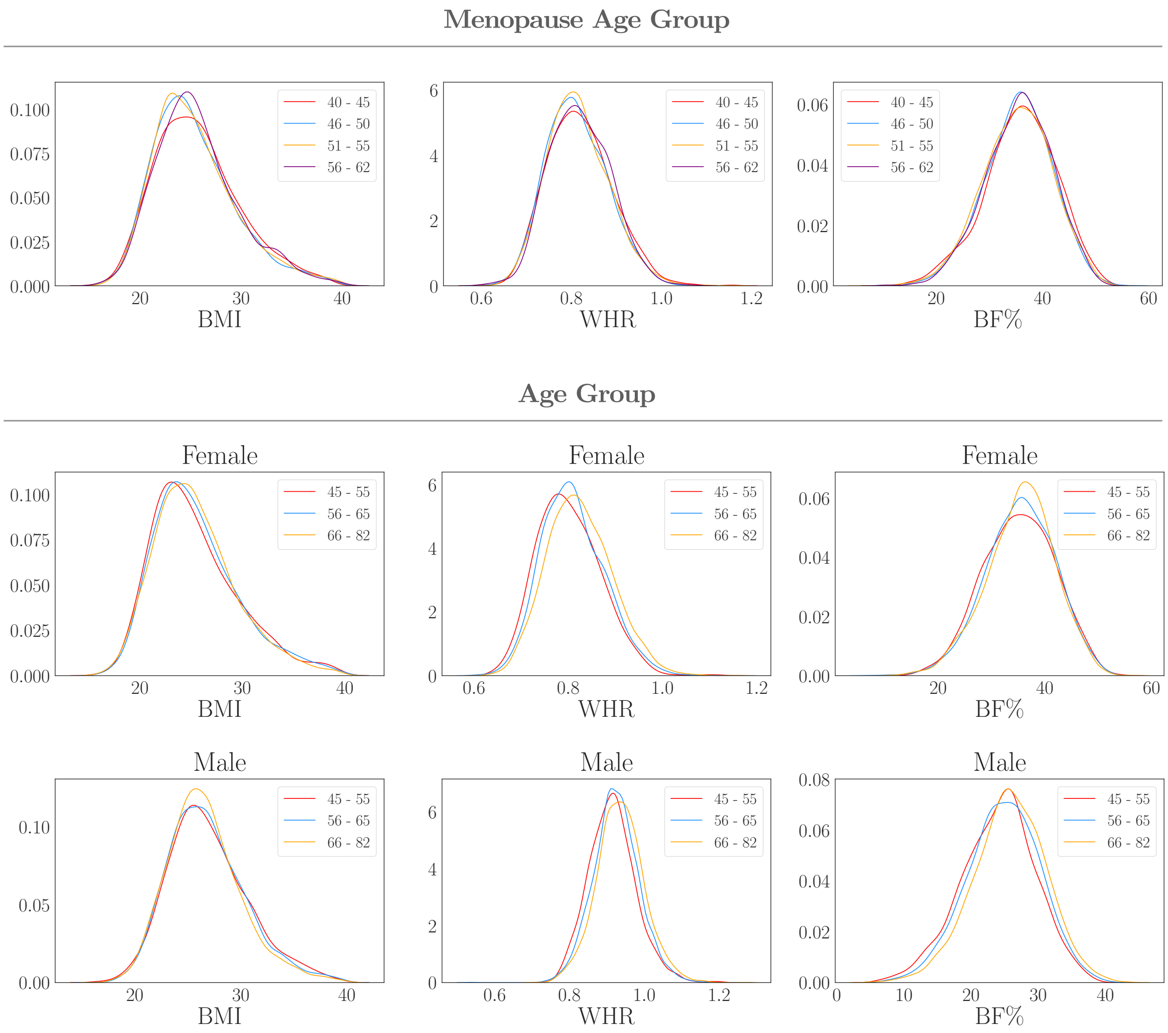

SI Figure 2: Distributions for body mass index (BMI), waist-to-hip ratio (WHR), and body fat percentage (BF%) within each Menopause Age group and each Age Group (see manuscript Table 2 for N in each group). The distributions are normalised to have the same area, and the y-axes represent the density.

3. Supplementary results for manuscript section 3.2

SI Table 4: Additional regression results based on formulae 1 and 2 in Section 2.7, including main effects of sex, CMR measures, and *APOE* status, and 3-way interactions between these variables. The dependent variable represents BAG values based on the mixed-sex age prediction model. Degrees of freedom for the main effects = 1, 21303 (formula 1), for the 3-way interactions = 1, 21299 (formula 2).

**Term**	***F***	***p***
Sex*	140.97	$< 2.2 \times 10^{-16}$
BMI	12.88	0.0003
WHR	162.68	$< 2.2 \times 10^{-16}$
BF%	0.78	0.38
APOE status	2.69	0.10
Sex × BMI × *APOE* status	0.01	0.91
Sex × WHR × *APOE* status	0.04	0.85
Sex × BF% × *APOE* status	1.73	0.19

*The main effect for Sex is reported based on the regression including BMI as CMR measure. This effect was consistent in the model using BFP (F value = 99.10) but not WHR (F = 1.11).)

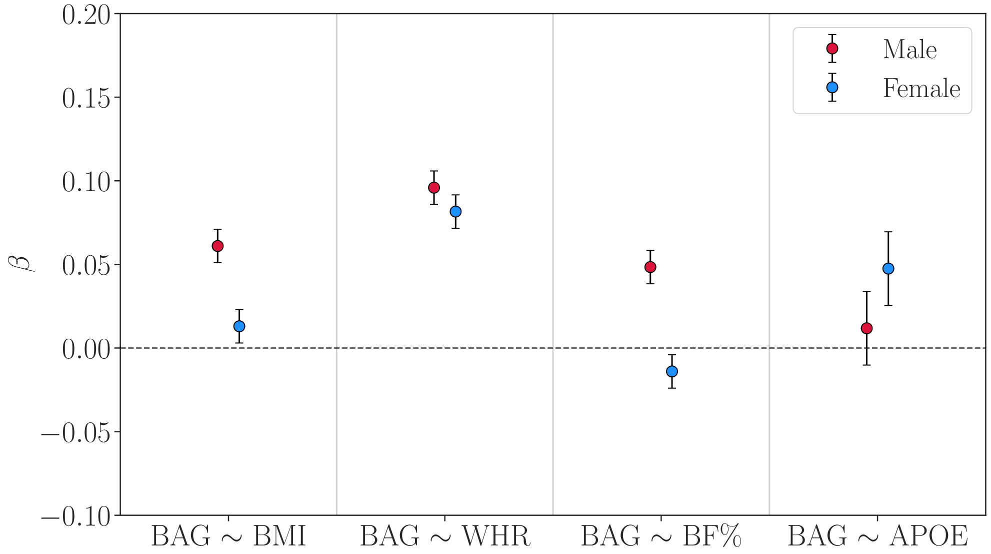

SI Figure 3: Associations between WM Brain Age Gap (BAG) based on the sex-specific models and each cardiometabolic factor as well as *APOE* status shown for males and females. β (y-axis) represents the beta value (slope) for each association, e.g., a positive β value indicates an association between greater cardiometabolic measures or *APOE4+* and higher BAG (older brain age relative to chronological age). The error bars represent standard errors on the β. BMI = body mass index, WHR = waist-to-hip ratio, BF% = body fat percentage.

4. Supplementary results for manuscript section 3.3.1

SI Table 5: Additional regression results based on formula 4 in Section 2.7, including main effects of Menopause Age Group and interactions between *APOE* status, CMR measures, and Menopause Age Group. The dependent variable represents BAG values based on the mixed-sex age prediction model. Degrees of freedom for the 3-way interactions = 3, 8753.

**Term**	***F***	***p***
Menopause Age Group*	5.21	0.001
BMI × *APOE* status × Menopause Age Group	0.29	0.83
WHR × *APOE* status × Menopause Age Group	0.63	0.60
BP% × *APOE* status × Menopause Age Group	0.51	0.68

*The main effect for Menopause Age Group is reported based on the regression including BMI as CMR measure. The results were consistent across all regressions run (F values between 5.20 and 5.35).

SI Table 6: Effects of interactions between the continuous variable Age at Menopause and BMI, WHR, BP% and *APOE* status on Brain Age Gap in females. Degrees of freedom = (1, 8765).

**Interaction**	***F***	***p***	***pcorr***
BMI × Menopause Age	1.15	0.28	0.38
WHR × Menopause Age	1.30	0.25	0.38
BF% × Menopause Age	0.78	0.38	0.38
APOE × Menopause Age	1.18	0.28	0.38

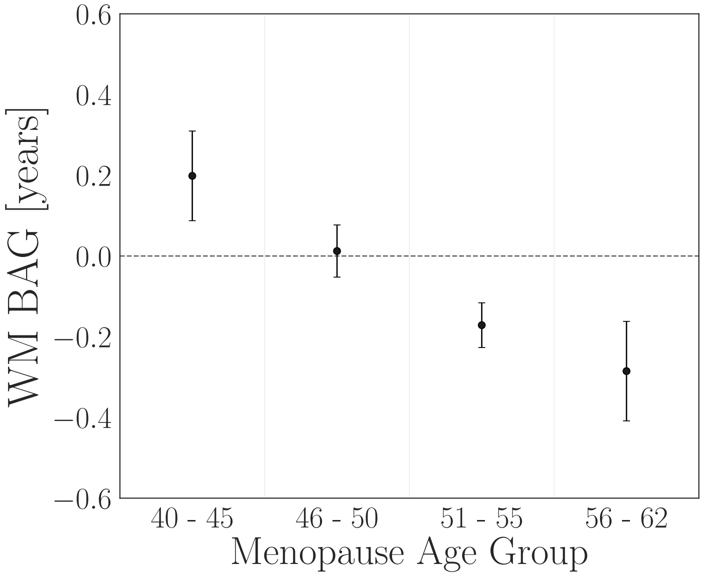

SI Figure 4: Mean white matter Brain Age Gap (WM BAG) plotted within each Menopause Age Group in females. Error bars represent the standard error on the mean.

SI Table 7: The interaction between Menopause Age Group and body mass index (BMI), waist-to-hip-ratio (WHR), body fat percentage (BF%), and *APOE4* status on BAG in females (formula 3, Section 2.7). This sample consisted of female participants included in the primary analyses in addition to those excluded due to early or late menopause, hysterectomy and/or oophorectomy (n = 9693). Degrees of freedom = (3, 9684).

**Interaction**	***F***	***p***	***pcorr***
BMI × Menopause Age Group	0.63	0.60	0.60
WHR × Menopause Age Group	1.48	0.22	0.60
$BF\%$ × Menopause Age Group	0.85	0.47	0.60
$APOE$ × Menopause Age Group	0.65	0.58	0.60

SI Table 8: The interaction between Age Group and BMI, WHR, BF%, and *APOE* genotype on BAG in females (formula 5, Section 2.7). This sample consisted of female participants included in the primary analyses in addition to those excluded due to early or late menopause, hysterectomy and/or oophorectomy (n = 9693). Degrees of freedom = (2, 9687).

**Interaction**	***F***	***p***	***pcorr***
BMI × Age Group	0.78	0.46	0.61
WHR × Age Group	2.08	0.13	0.50
$BF\%$ × Age Group	1.05	0.35	0.61
$APOE$ × Age Group	0.30	0.74	0.74

SI Table 9: Regression results based on formula 7 in Section 2.7, including main effects and interactions between *APOE* status and CMR measures within each Age Group in females. This sample consisted of female participants included in the primary analyses in addition to those excluded due to early or late menopause, hysterectomy and/or oophorectomy (n = 9693). Degrees of freedom: 45-55yrs (1, 1093), 56-65yrs (1, 4412), 66-82yrs (1, 4173) across main effects and 2-way interactions.

**45-55yrs**			
**Term**	***F***	***p***	***pcorr***
BMI	0.70	0.40	0.64
WHR	2.04	0.15	0.47
$BF\%$	0.35	0.55	0.64
APOE status*	2.37	0.12	0.47
BMI $\times APOE$ status	0.12	0.73	0.73
WHR $\times APOE$ status	0.46	0.50	0.64
$BF\%$ $\times APOE$ status	1.65	0.20	0.47
**56-65yrs**			
**Term**	***F***	***p***	***pcorr***
BMI	0.24	0.62	0.87
WHR	15.14	$\mathbf{1.00 \times 10^{-4}}$	$\mathbf{7.00 \times 10^{-4}}$
$BF\%$	1.71	0.19	0.55
APOE status*	1.39	0.24	0.55
BMI $\times APOE$ status	0.02	0.88	0.88
WHR $\times APOE$ status	0.72	0.40	0.69
$BF\%$ $\times APOE$ status	0.02	0.87	0.88
**66-82yrs**			
**Term**	***F***	***p***	***pcorr***
BMI	0.72	0.40	0.46
WHR	29.96	$\mathbf{4.66 \times 10^{-8}}$	$\mathbf{3.26 \times 10^{-7}}$
$BF\%$	4.63	**0.03**	0.11
APOE status*	1.50	0.22	0.31
BMI $\times APOE$ status	1.54	0.22	0.31
WHR $\times APOE$ status	0.38	0.54	0.54
$BF\%$ $\times APOE$ status	2.14	0.14	0.31

*The main effects for *APOE* status are reported based on the regressions including BMI as CMR measure. The results were consistent across all regressions run.

5. Supplementary results for manuscript section 3.3.2

SI Table 10: Additional regression results based on formula 6 in Section 2.7, including interactions between *APOE* status, CMR measures, and Age Group. Degrees of freedom for males (2, 10593) and females (2, 8758) across the 3-way interactions.

	Male		Female	
**Term**	F	p	F	p
BMI × Age Group × *APOE* status	0.64	0.53	0.35	0.71
WHR × Age Group × *APOE* status	0.15	0.86	0.04	0.97
BF% × Age Group × *APOE* status	0.98	0.38	1.10	0.33

SI Table 11: Effects of interactions between the continuous variable Age and BMI, WHR, BP% and *APOE* status on Brain Age Gap. Degrees of freedom for males (1, 10601) and females (1, 8766).

	Male			Female		
**Term**	F	p	$pcorr$	F	p	$pcorr$
BMI × Age	2.48	0.12	0.23	2.89	0.09	0.18
WHR × Age	0.13	0.72	0.81	5.41	0.02	0.08
BF% × Age	0.06	0.81	0.81	1.97	0.16	0.21
APOE × Age	2.49	0.11	0.23	0.39	0.53	0.53

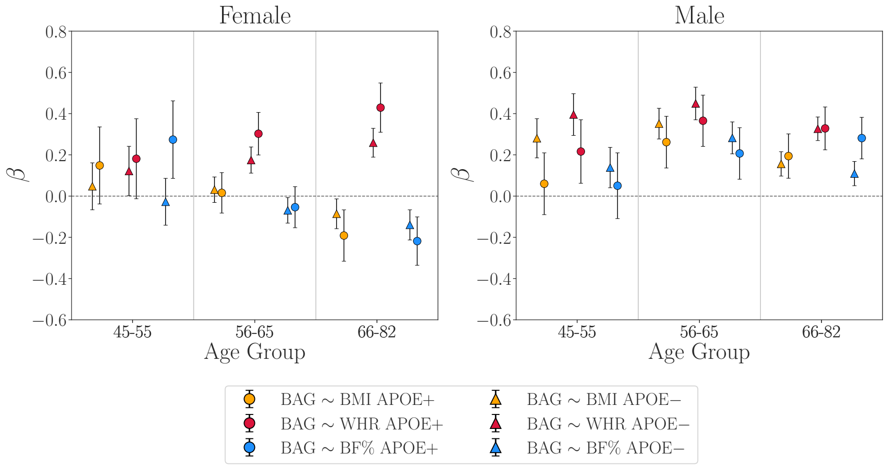

SI Figure 5: Associations between WM BAG and cardiometabolic risk factors grouped by *APOE* status (+ for carriers, − for non-carriers) within each Age Group bin. β (y-axis) represents the beta value (slope) for each association. The error bars represent standard errors on the β. BMI = body mass index, WHR waist-to-hip ratio, BF% = body fat percentage.

SI Table 12: Regression results based on formula 7 in Section 2.7, including main effects and interactions between *APOE* status and CMR measures within each Age Group separately in females and males. Degrees of freedom for men: 45-55yrs (1, 1646), 56-65yrs (1, 3711), 66-82yrs (1, 5233) and for females: 45-55yrs (1, 1044), 56-65yrs (1, 4122), 66-82yrs (1, 3589) across main effects and 2-way interactions.

	Male			Female		
**45-55yrs**						
**Term**	***F***	***p***	***pcorr***	***F***	***p***	***pcorr***
BMI	6.87	**0.009**	**0.03**	0.65	0.42	0.71
WHR	15.94	$\mathbf{6.81 \times 10^{-5}}$	$\mathbf{5.00 \times 10^{-4}}$	1.86	0.17	0.40
$BF\%$	1.73	0.19	0.33	0.44	0.51	0.71
APOE status*	0.70	0.40	0.56	2.34	0.13	0.40
BMI ×*APOE* status	1.71	0.19	0.33	0.26	0.61	0.71
WHR ×*APOE* status	0.44	0.51	0.59	0.10	0.75	0.75
$BF\%$ ×*APOE* status	0.12	0.73	0.73	2.08	0.15	0.40
**56-65yrs**						
**Term**	***F***	***p***	***pcorr***	***F***	***p***	***pcorr***
BMI	26.14	$\mathbf{3.34 \times 10^{-7}}$	$\mathbf{1.17 \times 10^{-6}}$	0.26	0.61	0.86
WHR	40.44	$\mathbf{2.27 \times 10^{-10}}$	$\mathbf{1.59 \times 10^{-9}}$	15.14	$\mathbf{1.00 \times 10^{-4}}$	$\mathbf{7.00 \times 10^{-4}}$
$BF\%$	15.74	$\mathbf{7.41 \times 10^{-5}}$	$\mathbf{1.73 \times 10^{-4}}$	1.53	0.22	0.51
APOE status*	0.04	0.85	0.85	1.35	0.24	0.51
BMI ×*APOE* status	0.42	0.52	0.72	0.02	0.89	0.90
WHR ×*APOE* status	0.32	0.57	0.72	1.13	0.29	0.51
$BF\%$ ×*APOE* status	0.25	0.62	0.72	0.02	0.90	0.90
**66-82yrs**						
**Term**	***F***	***p***	***pcorr***	***F***	***p***	***pcorr***
BMI	10.12	**0.001**	**0.005**	3.22	0.07	0.17
WHR	42.61	$\mathbf{7.30 \times 10^{-11}}$	$\mathbf{5.11 \times 10^{-10}}$	24.98	$\mathbf{6.06 \times 10^{-7}}$	$\mathbf{4.2 \times 10^{-6}}$
$BF\%$	9.00	**0.003**	**0.006**	6.76	**0.009**	**0.03**
APOE status*	1.60	0.21	0.29	0.96	0.33	0.46
BMI ×*APOE* status	0.08	0.78	0.91	0.53	0.47	0.54
WHR ×*APOE* status	0.001	0.97	0.97	1.52	0.22	0.38
$BF\%$ ×*APOE* status	2.19	0.14	0.24	0.32	0.57	0.57

*The main effects for *APOE* status are reported based on the regressions including BMI as CMR measure. The results were consistent across all regressions run.

6. Sensitivity analyses - manuscript section 3.3.2

The regression analyses testing associations within each Age Group (Section 3.3.) were re-run to account for potential confounding factors. In both females and males, we included the following covariates (in addition to age) based on previous studies indicating that these factors are associated with brain characteristics or cardiometabolic health: socioeconomic factors [4, 5, 6], including *educational level* (see manuscript Table 1 and `https://biobank.ndph.ox.ac.uk/showcase/field.cgi?id=6138`), *income* (annual £), and *Townsend Deprivation Index* [7, 8], a measure of material deprivation based on census data, *alcohol intake* [9, 10] (units per week/month), and *physical activity* [11, 12] (the number of days of moderate/vigorous physical activity for at least 10 minutes). For females, we also included *age at menopause* [13], *number of previous childbirths* [14], and *hormone replacement therapy use* (user versus never-user) [15, 16]. The results were consistent with the main analyses, as shown in SI Table 13.

SI Table 13: Regression results based on formula 7 in Section 2.7, including main effects and interactions between *APOE* status and CMR measures within each Age Group separately in males and females. Degrees of freedom for males: 45-55yrs (1,1619), 56-65yrs (1, 3684), 66-82yrs (1, 5206) and for females: 45-55yrs (1, 1014), 56-65yrs (1, 4092), 66-82yrs (1, 3559) across main effects and 2-way interactions.

	Male			Female		
**45-55yrs**						
**Term**	***F***	***p***	***pcorr***	***F***	***p***	***pcorr***
BMI	9.38	**0.002**	**0.008**	0.35	0.56	0.69
WHR	18.94	$\mathbf{1.44 \times 10^{-5}}$	$\mathbf{1.00 \times 10^{-4}}$	1.15	0.28	0.66
$BF\%$	3.97	**0.05**	0.11	0.23	0.63	0.69
APOE status*	0.77	0.38	0.44	1.86	0.17	0.61
BMI ×*APOE* status	2.37	0.12	0.22	0.16	0.69	0.69
WHR ×*APOE* status	0.80	0.37	0.44	0.28	0.60	0.69
$BF\%$ ×*APOE* status	0.23	0.63	0.63	1.93	0.16	0.61
**56-65yrs**						
**Term**	***F***	***p***	***pcorr***	***F***	***p***	***pcorr***
BMI	24.77	$\mathbf{6.76 \times 10^{-7}}$	$\mathbf{2.36 \times 10^{-6}}$	0.65	0.42	0.59
WHR	36.69	$\mathbf{1.53 \times 10^{-9}}$	$\mathbf{1.10 \times 10^{-8}}$	16.37	$\mathbf{5.31 \times 10^{-5}}$	$\mathbf{4.00 \times 10^{-4}}$
$BF\%$	13.86	$\mathbf{2.00 \times 10^{-4}}$	$\mathbf{5.00 \times 10^{-4}}$	1.19	0.27	0.51
APOE status*	0.0002	0.99	0.99	1.11	0.29	0.51
BMI ×*APOE* status	0.36	0.55	0.71	0.01	0.92	0.92
WHR ×*APOE* status	0.26	0.61	0.71	1.18	0.28	0.51
$BF\%$ ×*APOE* status	0.44	0.51	0.71	0.02	0.88	0.92
**66-82yrs**						
**Term**	***F***	***p***	***pcorr***	***F***	***p***	***pcorr***
BMI	9.02	**0.003**	**0.009**	1.17	0.28	0.40
WHR	39.11	$\mathbf{4.33 \times 10^{-10}}$	$\mathbf{3.0 \times 10^{-9}}$	24.95	$\mathbf{6.17 \times 10^{-7}}$	$\mathbf{4.31 \times 10^{-6}}$
$BF\%$	7.68	**0.006**	**0.01**	4.28	**0.04**	0.14
APOE status*	1.31	0.25	0.35	1.15	0.28	0.40
BMI ×*APOE* status	0.10	0.75	0.87	0.54	0.46	0.54
WHR ×*APOE* status	0.009	0.92	0.92	1.16	0.28	0.40
$BF\%$ ×*APOE* status	2.22	0.14	0.20	0.37	0.54	0.54

*The main effects for *APOE* status are reported based on the regressions including BMI as CMR measure. The results were consistent across all regressions run.

7. Differences (Z) in BAG ~ CMR associations in females - manuscript section 3.3.2

The follow-up regression analyses within each age group (manuscript Figure 3, SI Table 8 and 9) indicated that the divergence between the BAG associations with WHR versus BMI/BF% increased with older age in females. As a post-hoc test, we estimated the differences between the WHR vs. BMI/BF% associations for females within each age group in a pairwise manner using a Z test for correlated samples [17]:

$$Z = (\beta_{\text{WHR}} - \beta)/\sqrt{\sigma^2_{\text{WHR}} + \sigma^2 - 2\rho\sigma_{\text{WHR}}\sigma}\,. \quad (1)$$

In formula 1, β_{WHR} = beta value from the linear regression between BAG and WHR, β = beta value from the linear regression between BAG and BMI or BF%; σ = standard errors of the corresponding beta coefficients; ρ = the correlation between WHR and BMI or BF%. The results confirmed that the divergence (Z) between the associations with WHR versus BMI/BF% increased with age, and was most prominent in the oldest age group (SI Table 10).

SI Table 14: Differences in BAG associations (β) with WHR versus BAG associations with BMI/BF% for each age group in females (z-test for correlated samples; formula 1).

	45-55yrs			56-65yrs			66-82yrs		
	Z	***p***	***pcorr***	***Z***	***p***	***pcorr***	***Z***	***p***	***pcorr***
WHR vs. BMI	0.508	0.611	0.918	3.248	0.001	0.003	5.966	< 0.001	< 0.001
WHR vs. BF%	0.720	0.472	0.918	4.481	< 0.001	< 0.001	6.797	< 0.001	< 0.001

References

[1] P. J. Basser, J. Mattiello, D. LeBihan, MR diffusion tensor spectroscopy and imaging, Biophysical journal 66 (1994) 259–267.

[2] J. H. Jensen, J. A. Helpern, A. Ramani, H. Lu, K. Kaczynski, Diffusional kurtosis imaging: the quantification of non-gaussian water diffusion by means of magnetic resonance imaging, Magnetic Resonance in Medicine: An Official Journal of the International Society for Magnetic Resonance in Medicine 53 (2005) 1432–1440.

[3] E. Fieremans, J. H. Jensen, J. A. Helpern, White matter characterization with diffusional kurtosis imaging, Neuroimage 58 (2011) 177–188.

[4] A. Steptoe, P. Zaninotto, Lower socioeconomic status and the acceleration of aging: An outcome-wide analysis, Proceedings of the National Academy of Sciences 117 (2020) 14911–14917.

[5] K. B. Walhovd, A. M. Fjell, Y. Wang, I. K. Amlien, A. M. Mowinckel, U. Lindenberger, S. Düzel, D. Bartrés-Faz, K. P. Ebmeier, C. A. Drevon, et al., Education and income show heterogeneous relationships to lifespan brain and cognitive differences across european and us cohorts. (2021).

[6] A. F. Fotenos, M. A. Mintun, A. Z. Snyder, J. C. Morris, R. L. Buckner, Brain volume decline in aging: evidence for a relation between socioeconomic status, preclinical alzheimer disease, and reserve, Archives of neurology 65 (2008) 113–120.

[7] P. Townsend, Deprivation, Journal of social policy 16 (1987) 125–146.

[8] P. Townsend, P. Phillimore, A. Beattie, Health and deprivation: inequality and the North, Routledge, 1988.

[9] A. Topiwala, C. L. Allan, V. Valkanova, E. Zsoldos, N. Filippini, C. Sexton, A. Mahmood, P. Fooks, A. Singh-Manoux, C. E. Mackay, et al., Moderate alcohol consumption as risk factor for adverse brain outcomes and cognitive decline: longitudinal cohort study, bmj 357 (2017) j2353.

[10] A. Z. Fan, M. Russell, S. Stranges, J. Dorn, M. Trevisan, Association of lifetime alcohol drinking trajectories with cardiometabolic risk, The Journal of Clinical Endocrinology & Metabolism 93 (2008) 154–161.

[11] D. A. Raichlen, Y. C. Klimentidis, P. K. Bharadwaj, G. E. Alexander, Differential associations of engagement in physical activity and estimated cardiorespiratory fitness with brain volume in middle-aged to older adults, Brain imaging and behavior 14 (2020) 1994–2003.

[12] C. Phillips, Lifestyle modulators of neuroplasticity: how physical activity, mental engagement, and diet promote cognitive health during aging., Neural plasticity (2017).

[13] R. D. Brinton, J. Yao, F. Yin, W. J. Mack, E. Cadenas, Perimenopause as a neurological transition state, Nature reviews endocrinology 11 (2015) 393–405.

[14] I. Voldsbekk, C. Barth, I. I. Maximov, T. Kaufmann, D. Beck, G. Richard, T. Moberget, L. T. Westlye, A.-M. G. de Lange, A history of previous childbirths is linked to women's white matter brain age in midlife and older age, Human Brain Mapping (2021).

[15] E. Hogervorst, J. Williams, M. Budge, W. Riedel, J. Jolles, The nature of the effect of female gonadal hormone replacement therapy on cognitive function in post-menopausal women: a meta-analysis, Neuroscience 101 (2000) 485–512.

[16] P. M. Maki, L. Dennerstein, M. Clark, J. Guthrie, P. LaMontagne, D. Fornelli, D. Little, V. W. Henderson, S. M. Resnick, Perimenopausal use of hormone therapy is associated with enhanced memory and hippocampal function later in life, Brain research 1379 (2011) 232–243.

[17] D. W. Zimmerman, Correcting two-sample" z" and" t" tests for correlation: An alternative to one-sample tests on difference scores., Psicologica: International Journal of Methodology and Experimental Psychology 33 (2012) 391–418.
